# A cross-cultural examination of ethical reasoning in legal education: the interplay of critical thinking, case analysis skills, and professional identity formation

**DOI:** 10.3389/fpsyg.2026.1880466

**Published:** 2026-08-19

**Authors:** Xin Yuan

**Affiliations:** School of Law, Tiangong University, Tianjin, China

**Keywords:** case analysis skills, critical thinking, cross-cultural study, ethical reasoning, legal education, professional identity formation

## Abstract

**Introduction:**

Given the increasing emphasis on ethical competence in legal professions, this study investigates how legal education fosters ethical reasoning among law students through a serial moderated mediation model.

**Methods:**

Drawing on face-to-face survey data from 549 law students in China (*n* = 286) and South Korea (*n* = 263), the study explores the sequential mediating roles of case analysis skills and professional identity formation, as well as the moderating role of critical thinking. The proposed model was analyzed using partial least squares structural equation modeling (PLS-SEM) and multigroup analysis (MGA).

**Results:**

The findings reveal that legal education has a significant direct effect on ethical reasoning (β = 0.579, *p* < 0.001) and a significant serial indirect effect through case analysis skills and professional identity formation (β = 0.018, *p* = 0.008). Moreover, critical thinking amplifies the effect of legal education on analytical skill development and the influence of those skills on identity formation. Multigroup analysis further revealed significant cross-cultural differences: the direct effect of legal education on ethical reasoning was stronger among South Korean students, whereas the indirect and serial mediation pathways were stronger among Chinese students.

**Discussion:**

These results contribute to theoretical models of professional ethics and legal instruction and offer practical guidance for designing culturally responsive legal education curricula that promote ethical engagement.

## Introduction

The growing complexity of legal systems and the rise of socio-ethical challenges in contemporary societies have heightened the demand for ethically grounded legal professionals. Despite the widespread institutionalization of legal education across jurisdictions, questions remain regarding the factors and developmental mechanisms through which legal education is associated with ethical reasoning among future lawyers (Shi et al., 2024). Reports from professional bodies and academic evaluations alike suggest that students often graduate with technical proficiency but without a fully developed

ethical compass (Fedele et al., 2024). This raises an urgent question: can legal education meaningfully contribute to students' ethical development, or does it merely transmit procedural knowledge and doctrinal clarity?

Recent shifts in educational psychology (Prendeville and Kinsella, 2022) and legal pedagogy (Lewis and Stoyanovich, 2022) emphasize that ethical reasoning (Andersson et al., 2022; Stefkovich and Frick, 2021) is not solely the product of theoretical instruction, but rather the outcome of a more intricate developmental process involving analytical skill-building and identity formation. However, not all students experience this developmental trajectory uniformly. Some transition from legal education into professional roles with a clear ethical framework, while others struggle to internalize the responsibilities tied to legal practice (Lewis and Stoyanovich, 2022). This variation raises questions about the mechanisms linking legal instruction to ethical development and the role of critical thinking in shaping this process. Although educational interventions have been associated with students' ethical reasoning and related behavior (Prashar et al., 2024), existing evidence provides less clarity regarding the ordered psychological processes through which legal instruction becomes ethically meaningful.

Prior research has largely examined legal curricula, professional codes, and direct educational outcomes, while providing limited explanation of how legal education is translated into analytical competence, professional identity formation, and ethical reasoning (Hamilton and Monson, 2012; Nurutdinova et al., 2021; Treviño et al., 2006). Previous studies have generally considered these outcomes separately rather than examining whether analytical competence and professional identity formation operate as connected developmental mechanisms. The proposed model addresses this limitation by drawing on situated learning theory, social cognitive theory, professional identity theory, and cognitive moral development theory. Situated learning theory suggests that engagement with authentic professional tasks develops context-specific competence (Lave and Wenger, 1991), while social cognitive theory explains how educational environments and individual cognitive characteristics jointly shape learning outcomes (Bandura, 1986). These perspectives support the positioning of case analysis skills as an immediate outcome of legal education and critical thinking as a condition that may strengthen or weaken this relationship. Evidence from Chinese legal education similarly indicates that situational and interactive teaching can support students' learning effectiveness by engaging them more actively with contextualized legal material (Shi et al., 2024). The importance of complex logical reasoning in processing multifaceted information further supports the inclusion of case analysis skills in the model (Jin et al., 2026).

Professional identity theory suggests that participation in professional activities helps individuals internalize professional roles, values, and responsibilities (Cruess et al., 2015; Ibarra, 1999; Wald, 2015). Research across legal and other professional education settings further indicates that professional identity develops through guided participation, mentoring, instruction, reflection, and engagement with practice-based responsibilities (Hamilton, 2023a; Toh et al., 2022; Vabo et al., 2022). Cognitive moral development theory similarly proposes that ethical reasoning develops through engagement with complex moral problems and competing principles (Kohlberg, 1981; Rest, 1986). Professional identity formation has also been positively associated with levels of moral reasoning, suggesting that the internalization of professional roles and values is closely related to how students evaluate ethical issues (Sepahi et al., 2024). Accordingly, case analysis skills and professional identity formation are positioned as serial rather than parallel mediators: legal education first develops students' capacity to interpret legal problems, which then supports the internalization of professional values and, ultimately, ethical reasoning. A parallel model would treat case analysis skills and professional identity formation as independent mechanisms, whereas the proposed serial model recognizes that students' repeated engagement in legal analysis may provide the practice-based foundation through which a professional self-conception is subsequently formed. The sequence therefore represents a progression from analytical competence to professional identification and then to ethical reasoning (Dong et al., 2024). Critical thinking is included as a moderator because students differ in how deeply they evaluate legal information, question assumptions, and reflect on the professional implications of legal decisions (Appleby et al., 2013; James and Burton, 2017; Li and Sun, 2022). Thus, the model identifies both the developmental pathway and the cognitive condition through which legal education is associated with ethical reasoning.

Empirical examinations of these interconnected mechanisms remain limited in non-Western legal education contexts (Shi et al., 2024). Cultural and institutional settings may shape professional identity formation, reasoning practices, and responses to authority and ethical conflict. Cultural identity is continuously constructed through social and institutional contexts (Fan and Zhao, 2025), while culturally embedded capabilities may influence how individual competencies are translated into professional outcomes across national settings (Zhang et al., 2026). China and South Korea provide a meaningful comparison because both reflect East Asian educational traditions and rapidly developing legal education systems, while differing in their institutional structures, professional socialization practices, and approaches to legal training. In China, situational and technology-supported approaches are increasingly being incorporated into legal teaching to strengthen contextualized learning and student engagement (Shi et al., 2024). In South Korea, legal education reflects the interaction between reforms influenced by American legal education and continuing Confucian ethical orientations, illustrating how institutional training may coexist with culturally embedded professional values (Wu and Kim, 2022). This comparison therefore enables an assessment of whether the proposed pathways are consistent across the two settings or vary according to cultural and educational context.

**Figure 1:**
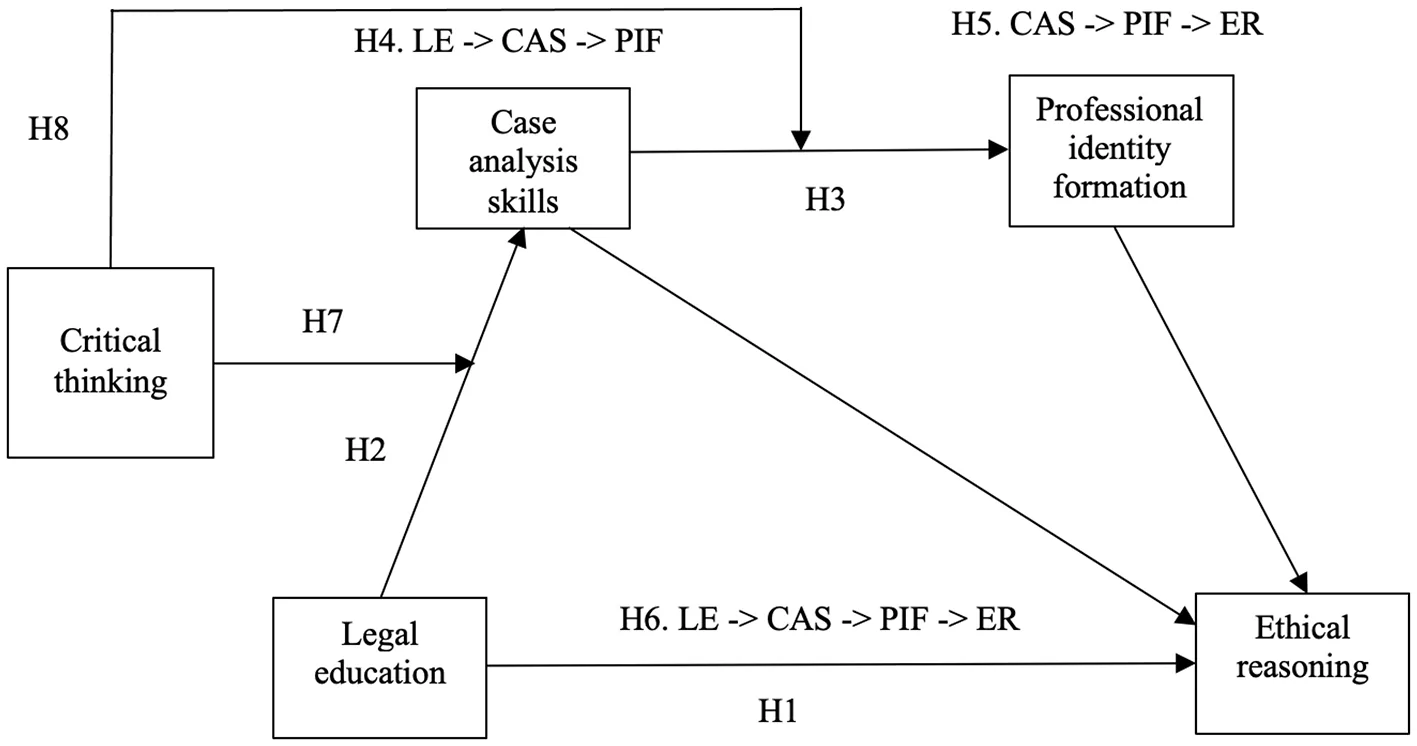
Proposed theoretical model.

This study contributes to the literature in three ways. First, it integrates cognitive, professional identity formation, and moral development perspectives to explain how legal education is associated with ethical reasoning through a theoretically ordered process. In doing so, it connects theoretical perspectives that have generally been applied separately to analytical learning, professional socialization, and moral development. Second, it tests serial mediation and moderation within a single structural model. More specifically, it distinguishes an ordered cognitive-to-identity pathway from parallel explanations that treat case analysis skills and professional identity formation as independent mechanisms. Third, it extends legal education research through a cross-cultural comparison of Chinese and South Korean law students. This comparison addresses the limited evidence regarding whether the same psychological mechanisms operate similarly across two culturally related but institutionally distinct East Asian legal education settings. Accordingly, the study examines how legal education is associated with ethical reasoning through case analysis skills and professional identity formation, while considering critical thinking and cultural context as important conditions of this developmental process (Figure 1).

## Hypotheses

### Direct effect of legal education on ethical reasoning

Legal education plays a fundamental role in shaping the ethical orientation of future legal professionals (Ajevski et al., 2023). It goes beyond transmitting legal rules and procedures by aiming to cultivate reasoning that aligns with professional and societal norms. Ethical reasoning, in this context, refers to the capacity to analyze, reflect upon, and act in accordance with moral and legal principles in complex scenarios (Stefkovich and Frick, 2021). While traditional law curricula have emphasized doctrinal knowledge, there is a growing consensus that legal education must also foster the moral and ethical sensibilities required for responsible practice (Dogan et al., 2024). This relationship can be explained through cognitive moral development theory, which proposes that moral reasoning develops as individuals engage with increasingly complex ethical dilemmas, evaluate competing principles, and move beyond simple reliance on externally imposed rules (Kohlberg, 1981; Rest, 1986). Legal education provides such developmental opportunities by exposing students to conflicting rights, professional obligations, legal ambiguities, and value-laden cases that require reasoned ethical reasoning. From a social cognitive perspective, the educational environment also shapes ethical reasoning through observation, guided practice, feedback, and repeated interaction with professional standards (Bandura, 1986).

Recent studies have highlighted that structured exposure to legal ethics, case-based dilemmas, and reflective discussion can significantly enhance students' ability to recognize ethical issues and reason through them (Herring, 2023). Evidence from higher education also indicates that sustained ethics-related instruction can strengthen students' ethical reasoning and corresponding behavior, reinforcing the view that educational experiences can contribute to ethical development beyond technical knowledge acquisition (Prashar et al., 2024). Moreover, students who perceive their legal education as robust and principled are more likely to report higher confidence in making ethically sound decisions in simulated or real-life situations (Young, 2023). This is especially crucial in today's legal environments, where ethical complexities are often entangled with ambiguous laws and sociopolitical pressures. Similarly, Xhemajli (2021) emphasized that ethical maturity develops when students are consistently challenged to reflect on the moral dimensions of law, suggesting that education structures can scaffold ethical growth.

Thus, the present study hypothesizes that:

*H1. Legal education is positively related to ethical reasoning*.

### Direct effect of legal education on case analysis skills

Legal education is designed not only to convey legal doctrines but also to develop the cognitive competencies essential for navigating complex legal problems (Ajevski et al., 2023). Among these, case analysis skills stand out as a core capability through which students learn to interpret precedents, evaluate arguments, and apply legal reasoning in practical contexts. These skills are cultivated through repeated engagement with case law, hypothetical scenarios, and classroom debates, all of which aim to sharpen students' abilities to identify key facts, apply relevant legal principles, and make well-supported legal arguments (Choi and Schwarcz, 2024). Situated learning theory provides a useful explanation for this relationship by proposing that competence develops through active participation in authentic and context-specific practices (Lave and Wenger, 1991). In legal education, repeated exposure to case analysis allows students to move beyond passive knowledge acquisition and engage directly with the interpretive and argumentative practices of the legal profession. Social cognitive theory further suggests that students develop analytical competencies through observation, guided practice, feedback, and repeated interaction with instructional models and professional standards (Bandura, 1986).

The acquisition of case analysis skills is often considered one of the primary learning outcomes of law school. As students progress through legal education, they are expected to move beyond rote memorization toward analytical thinking that allows for nuanced understanding and flexible application of legal frameworks. Research suggests that the structure and intensity of legal instruction significantly influence the development of these skills. For instance, Sokhanvar et al. (2021) found that students exposed to case-based teaching methods demonstrated higher levels of analytical precision and legal argumentation than those taught through traditional lecture formats. Likewise, Haynes et al. (2024) emphasized the importance of integrating real-world legal cases into classroom instruction to cultivate deeper engagement with legal reasoning. Related evidence from Chinese legal education shows that situational and interactive teaching can improve learning effectiveness by engaging students more actively with contextualized legal problems (Shi et al., 2024). Moreover, the theoretical underpinning of legal education as a form of cognitive apprenticeship supports the notion that students acquire analytical competencies by observing, practicing, and receiving feedback in the context of real legal challenges. The importance of complex logical reasoning in processing multifaceted information also reinforces the role of structured analytical training in developing case analysis competence (Jin et al., 2026).

Thus, it is proposed that:

*H2. Legal education is positively related to case analysis skills*.

### Direct effect of case analysis skills on professional identity formation

Professional identity formation is a central outcome of legal education, referring to how students internalize the roles, values, and responsibilities of the legal profession (Fines, 2025). It reflects the transition from being a student of the law to becoming a future legal practitioner who not only understands legal frameworks but also aligns with the ethical and professional norms of the field (Wald, 2015). While various factors contribute to identity development, one of the most important cognitive drivers is the acquisition of case analysis skills. Professional identity theory suggests that professional identities are gradually constructed through participation in professional practices, reflection on experiences, interaction with role models, and internalization of professional norms and values (Cruess et al., 2015; Ibarra, 1999; Wald, 2015). Rather than being acquired solely through classroom instruction, professional identity develops as students repeatedly engage in authentic disciplinary activities that shape how they perceive themselves as future members of the profession. Accordingly, case analysis provides an important mechanism through which students begin to think, reason, and behave like legal professionals.

Research on professional education supports this developmental logic. Guided participation, mentoring, instruction, and engagement with profession-specific responsibilities help learners internalize role expectations and construct a professional self-conception (Hamilton, 2023b; Toh et al., 2022). Similarly, practice-based learning and repeated opportunities to exercise professional judgment have been identified as central to professional identity development (Vabo et al., 2022). In legal education, case analysis skills may therefore contribute to identity formation by enabling students to engage with the interpretive, evaluative, and decision-making practices associated with legal work.

Empirical studies support this linkage. For instance, Tomlinson and Jackson (2021) found that students who performed well in legal reasoning and case analysis tasks were more likely to identify with professional values and express confidence in handling complex legal roles. Similarly, Stefkovich and Frick (2021) and Suarez and McGrath (2022) argued that analytical competence enhances students' ability to make ethically grounded judgments, which contributes to a stronger internalization of legal identity. These findings suggest that case analysis skills are not merely technical competencies; they also provide the experiential and reflective foundation through which students begin to internalize professional values, responsibilities, and role expectations. In essence, case analysis enables students to understand not just what the law is, but how it functions in practice—thus bridging the gap between intellectual knowledge and professional formation.

Therefore, this study posits the following:

*H3. Case analysis skills are positively related to professional identity formation*.

### Mediating role of case analysis skills

Hypotheses 1 and 2 suggest that legal education enhances students' case analysis skills, while analytical competence represents an essential foundation for higher-order legal reasoning and professional development. Drawing on situated learning theory and social cognitive theory, legal education is expected to be associated with ethical reasoning not only directly but also indirectly through the analytical competencies developed during legal training (Bandura, 1986; Lave and Wenger, 1991). As students repeatedly engage in case interpretation, legal argumentation, and problem-solving activities, they become better equipped to evaluate ethical dilemmas systematically and justify decisions using legal and moral principles. Case-based and situational learning therefore provides a cognitive pathway through which educational experiences are translated into more structured ethical evaluation (Choi and Schwarcz, 2024; Herring, 2023; Shi et al., 2024). Rather than assuming that exposure to legal instruction is sufficient by itself, this mediation argument recognizes that students must first develop the analytical capacity to identify relevant facts, compare competing claims, and evaluate the ethical implications of possible legal decisions.

Therefore, this study proposes the following:

H4. Case analysis skills mediate the relationship between legal education and ethical reasoning.

### Mediating role of professional identity formation

Building on Hypotheses 3 and 4, the study further expects that case analysis skills contribute to professional identity formation, which in turn is associated with ethical reasoning. Professional identity theory proposes that individuals who internalize the values, responsibilities, and norms of their profession are more likely to make decisions consistent with professional ethical standards (Cruess et al., 2015; Ibarra, 1999; Wald, 2015). Professional identity develops as learners interpret practice-based experiences, exercise professional judgment, and integrate occupational expectations into their emerging self-conceptions (Hamilton, 2023a; Toh et al., 2022; Vabo et al., 2022). In legal education, case analysis exposes students to the responsibilities, tensions, and value choices embedded in legal practice, thereby helping them understand not only how lawyers reason but also what professional obligations such reasoning entails.

As students increasingly perceive themselves as future members of the legal profession, they become more committed to upholding its ethical principles and responsibilities. Evidence of a positive association between professional identity formation and moral reasoning further suggests that the internalization of professional values corresponds with more developed ethical evaluation (Sepahi et al., 2024). Consequently, professional identity formation is expected to serve as an identity-based mechanism through which case analysis skills are associated with ethical reasoning.

Therefore, this study proposes the following:

H5. Professional identity formation mediates the relationship between case analysis skills and ethical reasoning.

### A serial mediation model

Building on the preceding relationships, the present study proposes that legal education is associated with ethical reasoning through an ordered developmental pathway. Specifically, legal education is expected to strengthen case analysis skills, which subsequently support professional identity formation and, ultimately, ethical reasoning. This sequence integrates cognitive skill development with identity internalization rather than treating them as unrelated mechanisms.

The proposed ordering is supported by the complementary functions of situated learning theory, professional identity theory, and cognitive moral development theory. Situated learning theory explains how participation in authentic legal tasks develops context-specific analytical competence (Lave and Wenger, 1991). Through repeated case interpretation, argument evaluation, and application of legal principles, students acquire the cognitive practices required for legal work (Shi et al., 2024; Zulela et al., 2022). Professional identity theory then explains how engagement in these disciplinary practices helps students internalize the values, responsibilities, and role expectations associated with the legal profession (Cruess et al., 2015; Ibarra, 1999; Wald, 2015). Research on professional identity formation similarly indicates that guided participation, instruction, reflection, and practice-based judgment provide important foundations for the development of a professional self-conception (Hamilton, 2023b; Toh et al., 2022; Vabo et al., 2022). Cognitive moral development theory further suggests that ethical reasoning advances as individuals confront complex dilemmas and evaluate competing principles through increasingly internalized standards (Kohlberg, 1981; Rest, 1986).

A parallel mediation model would imply that case analysis skills and professional identity formation independently transmit the relationship between legal education and ethical reasoning without a theoretically necessary connection between them. The present model instead proposes that the two mediators represent successive developmental stages. Case analysis skills constitute the more immediate cognitive outcome of legal instruction because students first learn how to interpret legal problems, weigh evidence, and construct reasoned conclusions through repeated engagement with contextualized legal tasks (Lave and Wenger, 1991; Shi et al., 2024). These analytical experiences then provide the practice-based foundation through which students begin to understand and internalize the norms, responsibilities, and ethical expectations of legal practice (Hamilton, 2023a; Toh et al., 2022; Vabo et al., 2022). Professional identity formation is therefore positioned downstream of analytical competence and more proximally to ethical reasoning.

This ordering does not suggest that case analysis skills and professional identity formation can never operate independently. Rather, it proposes that the full developmental mechanism is more adequately represented by a sequence in which competence enables meaningful participation in professional reasoning, and such participation facilitates identity internalization (Hamilton, 2023b; Toh et al., 2022). The reported positive association between professional identity formation and moral reasoning further supports the final link in this sequence (Sepahi et al., 2024). Although prior research has examined the individual relationships between educational participation, professional competence, identity formation, and ethical reasoning, the complete ordered pathway has received limited empirical examination in legal education. Testing serial mediation therefore represents a central contribution of the present study (Sheng et al., 2023).

Therefore, it is posited that case analysis skills and professional identity formation act as sequential mediators, together forming a bridge between legal education and ethical reasoning.

*H6. Case analysis skills and professional identity formation serially mediate the relationship between legal education and ethical reasoning*.

### Moderating role of critical thinking between legal education and case analysis skills

The moderating role of critical thinking in the relationship between legal education and case analysis skills is crucial to understanding how students differentially respond to instructional environments. Although legal education provides structured opportunities for students to develop analytical competencies, the extent to which students acquire and apply these skills may depend on their individual cognitive dispositions (Volokh, 2022). Critical thinking, as a reflective and evaluative thinking process, determines how learners process legal content, assess arguments, and internalize complex case-based reasoning (Li and Sun, 2022). Social cognitive theory provides a theoretical basis for this moderating role by proposing that learning outcomes emerge from the interaction between environmental inputs and individual cognitive characteristics (Bandura, 1986). Legal instruction may therefore offer similar learning opportunities to all students, but its contribution to case analysis skills may vary according to students' capacity to question assumptions, evaluate evidence, and integrate competing interpretations.

According to the elaboration likelihood model (Andre, 2025), individuals with high cognitive engagement are more likely to process information through a central route—systematically analyzing content and deriving deep meaning. In contrast, students with lower critical thinking tendencies may rely on superficial understanding, limiting their ability to fully develop case analysis skills even when exposed to the same educational stimuli. In this sense, critical thinking acts as a cognitive amplifier that intensifies the relationship between instructional input and analytical output (Appleby et al., 2013). Empirical evidence similarly suggests that students with stronger critical thinking engage more actively with complex learning materials, evaluate competing arguments more systematically, and demonstrate superior analytical performance in educational settings (Belladonna et al., 2025; James and Burton, 2017; Li and Sun, 2022). Consequently, students with higher critical thinking are expected to benefit more from legal instruction because they process case-based learning more deeply and apply legal concepts more systematically than students with lower critical thinking. Therefore, critical thinking may moderate the relationship between legal education and case analysis skills by either enhancing or attenuating the impact of legal instruction. When critical thinking is high, the positive effects of legal education on analytical skill development are likely to be stronger; when critical thinking is low, those effects may be weaker.

Thus, the following hypothesis is proposed:

*H7. Critical thinking moderates the relationship between legal education and case analysis skills, such that the relationship is stronger when critical thinking is higher*.

### Moderating role of critical thinking between case skills analysis and professional identity formation

Critical thinking is expected to moderate the relationship between case analysis skills and professional identity formation. While case analysis skills enable students to interpret legal texts, weigh arguments, and reason through complex scenarios, the extent to which these experiences contribute to professional identity formation may depend on students' critical thinking capacity. Critical thinking, defined as the ability to reflect, evaluate, and reason logically (Belladonna et al., 2025), equips learners to approach legal tasks not only with analytical precision but also with reflective awareness of their role within the broader legal system.

Social cognitive theory and professional identity theory jointly explain this moderating relationship. Social cognitive theory suggests that acquired competencies do not automatically produce uniform developmental outcomes because their effects depend partly on individuals' cognitive characteristics and reflective engagement (Bandura, 1986). Professional identity theory similarly proposes that professional identity develops when individuals interpret their experiences, evaluate professional expectations, and integrate those experiences into their emerging self-conceptions (Cruess et al., 2015; Ibarra, 1999; Wald, 2015). Thus, case analysis skills may provide the experiential foundation for identity formation, whereas critical thinking determines how deeply students interpret and internalize the professional implications of those experiences.

Drawing on the elaboration likelihood model (Andre, 2025), individuals with higher critical thinking are more likely to engage in deep, effortful processing of legal issues rather than relying on superficial cues. When students possess strong critical thinking abilities, their engagement with legal cases becomes a transformative experience, allowing them to internalize professional values more fully (Sarraf-Yazdi et al., 2021). In contrast, students with lower critical thinking may still develop technical proficiency in case analysis but may fail to connect those skills to a coherent professional identity, thereby limiting the developmental impact of their legal training. This argument is consistent with evidence that reflective reasoning and deliberate engagement with professional experiences support the internalization of professional values and role expectations (Hamilton, 2023a; Toh et al., 2022; Vabo et al., 2022). Accordingly, students with stronger critical thinking are expected to derive greater identity-related benefits from their analytical experiences because they are more likely to interpret legal reasoning through a professional and ethical lens. Therefore, this study posits that critical thinking strengthens the relationship between case analysis skills and professional identity formation. When critical thinking is high, students are more likely to process the implications of legal reasoning through a moral and professional lens, thereby enhancing the internalization of their future role as legal professionals.

*H8. Critical thinking moderates the relationship between case analysis skills and professional identity formation, such that the relationship is stronger when critical thinking is higher*.

Summary of proposed hypotheses is illustrated in Table 1.

**Table 1:** Summary of hypotheses.

Hypothesis	Proposed relationship
H1	Legal education is positively related to ethical reasoning.
H2	Legal education is positively related to case analysis skills.
H3	Case analysis skills are positively related to professional identity formation.
H4	Case analysis skills mediate the relationship between legal education and ethical reasoning.
H5	Professional identity formation mediates the relationship between case analysis skills and ethical reasoning.
H6	Case analysis skills and professional identity formation serially mediate the relationship between legal education and ethical reasoning.
H7	Critical thinking moderates the relationship between legal education and case analysis skills, such that the positive relationship is stronger when critical thinking is higher.
H8	Critical thinking moderates the relationship between case analysis skills and professional identity formation, such that the positive relationship is stronger when critical thinking is higher.

## Methodology

### Sample and sampling

This study employed a face-to-face, questionnaire-based data collection approach to examine how legal education influences ethical reasoning through the mediating roles of case analysis skills and professional identity formation, and the moderating role of critical thinking. Data were gathered from university law students in China and South Korea to enable a cross-cultural comparison of the proposed model. A structured survey instrument was administered in person to ensure comprehension of the questions, address any clarifications immediately, and enhance data quality. Face-to-face administration was particularly suitable in this context, as it encouraged more thoughtful participation and reduced the likelihood of incomplete responses.

Participants were approached within academic institutions, primarily during lecture breaks and tutorial sessions, where the study's objectives were briefly introduced. The data were collected from law students enrolled at 6 universities in China and 5 universities in South Korea, including both public and private institutions. The participating institutions were selected based on their provision of undergraduate or postgraduate legal education programs and their willingness to facilitate on-site data collection. Participation was voluntary, and respondents were assured of anonymity and confidentiality. Before distributing the survey, verbal and written consent was obtained from all participants. The study was reviewed and approved by the ethics committees of the host institutions in both countries. Students were informed that there were no right or wrong answers and that their honest input would contribute to research on legal education and professional development.

To improve the response rate, several steps were taken. First, permission was obtained from instructors and administrators to distribute the questionnaires at times that would not interfere with lectures. Second, data collection teams were trained to engage participants politely and professionally, explain the purpose of the study, and encourage full participation. Third, participants were given sufficient time (approximately 15–20 min) to complete the survey on-site, minimizing the chances of delayed or non-returned responses.

A purposive sampling technique was employed to target students who were enrolled in undergraduate or graduate legal education programs. This method was chosen due to its relevance in capturing individuals who were directly exposed to formal legal instruction and were therefore likely to be engaged in identity formation and ethical development processes. Given the model's focus on legal education outcomes, it was essential to sample participants from academic environments where legal curricula were actively delivered. Students from both public and private institutions were included to ensure diversity in academic exposure and institutional structure. The inclusion criteria required participants to be currently enrolled in an undergraduate or postgraduate law program, to have completed at least one semester of formal legal education, and to provide informed consent. Students who were not enrolled in a law program or submitted substantially incomplete questionnaires were excluded.

Data collection was carried out over a 3-month period in each context: from September to November 2023 in China and from December 2023 to February 2024 in South Korea. A total of 700 questionnaires were distributed; of which, 549 valid questionnaires were retained for the final analysis, comprising 286 responses from China and 263 responses from South Korea. The achieved sample exceeded the minimum recommendations for PLS-SEM and provided sufficient observations for estimating the proposed structural relationships and conducting multigroup comparisons (Hair et al., 2019). Each data collection session was supervised by the research team to ensure consistency in administration and to respond to any inquiries.

In China, approximately 53% of the respondents were female and 47% male, while in South Korea, 54% were female and 46% male. Regarding academic level, 70% of the Chinese participants were undergraduate law students and 30% were enrolled in postgraduate programs. Similarly, in South Korea, 66% were undergraduates and 34% were postgraduates. Age-wise, the Chinese sample comprised 10% aged 18–20, 49% aged 21–23, 32% aged 24–26, and 9% aged 27 or above. In South Korea, 14% were in the 18–20 age group, 45% were aged 21–23, 28% were aged 24–26, and 13% were aged 27 or above. Regarding institutional affiliation, 62% of the Chinese respondents were from public universities and 38% from private institutions, while in South Korea, 58% attended public institutions and 42% attended private institutions. The detailed demographics table is illustrated in Table 2.

**Table 2:** Demographic profile of the respondents.

Characteristic	Category	China (*n* = 286) (%)	South Korea (*n* = 263) (%)
Gender	Male	47	46
	Female	53	54
Academic level	Undergraduate	70	66
	Postgraduate	30	34
Age	18–20 years	10	14
	21–23 years	49	45
	24–26 years	32	28
	≥27 years	9	13
Institution type	Public university	62	58
	Private university	38	42

### Common method biasness

Given that all data were collected using a cross-sectional, self-report questionnaire, the potential for common method bias (CMB) was assessed using Harman's single-factor test. An unrotated exploratory factor analysis indicated that the first factor accounted for 34.27% of the total variance, which is below the commonly accepted threshold of 50% (Podsakoff et al., 2003). Therefore, common method bias was not considered a serious threat to the validity of the findings.

### Control variables

In this study, several control variables were included to account for individual and contextual factors that could potentially influence the relationships among legal education, case analysis skills, professional identity formation, critical thinking, and ethical reasoning. Specifically, gender, age, academic level (undergraduate vs. postgraduate), and type of institution (public vs. private) were treated as control variables in the structural model.

### Measures

All constructs were measured using validated scales from prior literature, adapted slightly to suit the legal education context. Items were rated on a 5-point Likert scale ranging from 1 (strongly disagree) to 5 (strongly agree), unless otherwise stated. Higher scores indicated greater levels of the measured construct. All scales demonstrated acceptable reliability and validity across the Chinese, South Korean, and combined samples, as reported in the psychometric analysis.

The questionnaire was initially prepared in English because the measurement scales were adopted from previously validated international studies. To ensure conceptual equivalence across the two study contexts, the instrument was translated independently into Chinese and Korean using the back-translation procedure recommended by Brislin (1980). Two bilingual experts translated the English questionnaire into each target language, after which independent bilingual translators, who were not involved in the initial translation, translated the questionnaires back into English. The original and back-translated versions were then compared, and any discrepancies in wording or meaning were discussed and resolved through consensus to ensure semantic and conceptual equivalence rather than literal translation. This procedure minimized potential semantic ambiguity and enhanced the cross-cultural comparability of the measurement instrument.

Before the main data collection, the adapted questionnaire was reviewed by two legal education scholars and one educational psychology expert to assess content relevance, clarity, and contextual appropriateness. A pilot test was then conducted with 30 law students, including participants from both cultural contexts. Minor wording adjustments were made based on their feedback, while no items were removed because the participants reported that the instructions and statements were clear and understandable.

### Legal education

Legal education was assessed using a 4-item scale adapted from Hamilton and Monson (2012), capturing students' perceptions of the quality and impact of their legal training. Sample items included statements such as “My legal education has prepared me to think critically about legal and ethical issues.”

### Critical thinking

Critical thinking was measured using a 6-item short form of the Critical Thinking Disposition Scale (Facione, 2011), reflecting students' habitual tendency to engage in reflective and analytical thought. Items assessed openness, reasoning, and evaluative skills, such as “I enjoy solving problems that require careful thought.”

### Case analysis skills

A 5-item scale was developed based on prior work by Schauer (2009) and Treviño et al. (2006), focusing on students' ability to interpret legal cases, identify arguments, and apply legal reasoning. A sample item is “I can break down legal cases into essential facts and legal principles.”

### Professional identity formation

This construct was measured using a 6-item scale adapted from Wald (2015) and Hamilton and Monson (2012), assessing how students internalize the values and roles of the legal profession. Sample items included “I see myself as a future legal professional with a clear sense of ethical responsibility.”

### Ethical reasoning

Ethical reasoning was measured using a 5-item scale adapted from Treviño et al. (2006), capturing students' ability to evaluate moral problems and justify ethical decisions in legal contexts. A sample item is “I can identify the ethical implications of legal decisions and arguments.”

## Results

The proposed model was analyzed using SmartPLS 4.0. Structural relationships were estimated using the partial least squares structural equation modeling (PLS-SEM) approach. Statistical significance was assessed through a non-parametric bootstrapping procedure with 5,000 bootstrap resamples, applying a two-tailed significance level of 5% (*p* < 0.05). The reliability and validity of the constructs were evaluated using Cronbach's alpha (α), composite reliability (CR), and average variance extracted (AVE) across the Chinese, South Korean, and combined samples (see, Table 3). All constructs demonstrated acceptable internal consistency, with Cronbach's alpha values exceeding the commonly recommended threshold of 0.70 (Nunnally and Bernstein, 1994), except for case analysis skills (CAS) and professional identity formation (PIF) in the Chinese sample, which had slightly lower alpha values of 0.720 and 0.700, respectively. However, their CR values were above 0.70, indicating adequate composite reliability (Hair et al., 2019). For the AVE, values across all constructs and samples were above 0.50, confirming convergent validity as per Fornell and Larcker's (1981) criterion. Specifically, critical thinking showed consistent psychometric properties in both cultural contexts, and legal education and ethical reasoning performed strongly across all three assessments. The combined sample revealed generally stronger psychometric indicators, supporting the robustness and consistency of the measurement model across contexts.

**Table 3:** Reliability and validity assessment (psychometric properties of constructs).

Constructs	China			South Korea			Complete		
	Alpha	CR	AVE	Alpha	CR	AVE	Alpha	CR	AVE
CAS	0.720	0.723	0.547	0.744	0.865	0.592	0.771	0.807	0.607
CT	0.779	0.779	0.531	0.819	0.831	0.576	0.815	0.816	0.574
ER	0.772	0.786	0.597	0.783	0.836	0.616	0.783	0.816	0.612
LE	0.758	0.765	0.580	0.826	0.828	0.658	0.801	0.804	0.627
PIF	0.700	0.705	0.617	0.784	0.796	0.697	0.744	0.752	0.661

CAS, case analysis skills; CT, critical thinking; ER, ethical reasoning; LE, legal education; PIF, professional identity formation.

**Figure 2:**
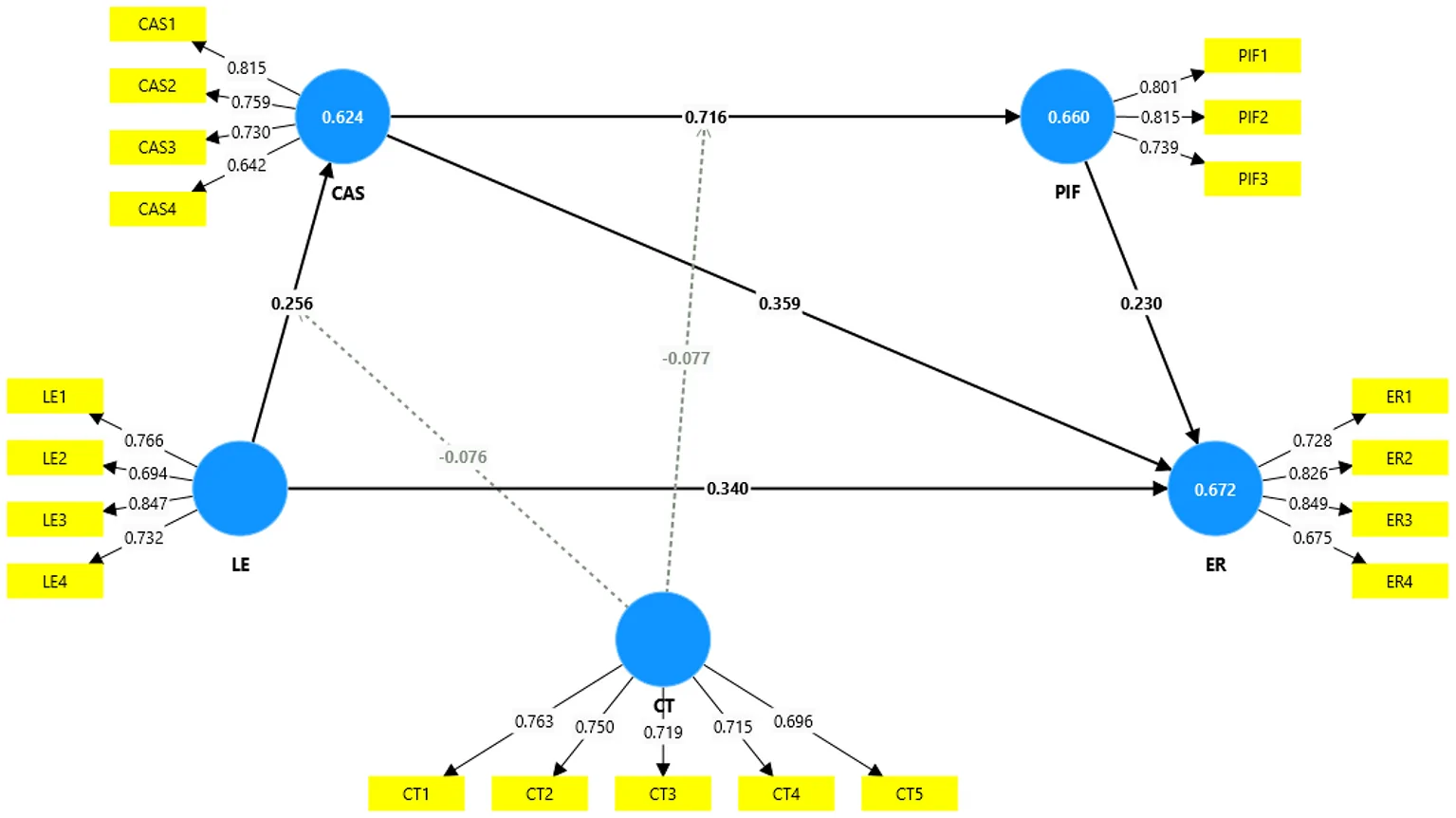
SEM (China).

**Figure 3:**
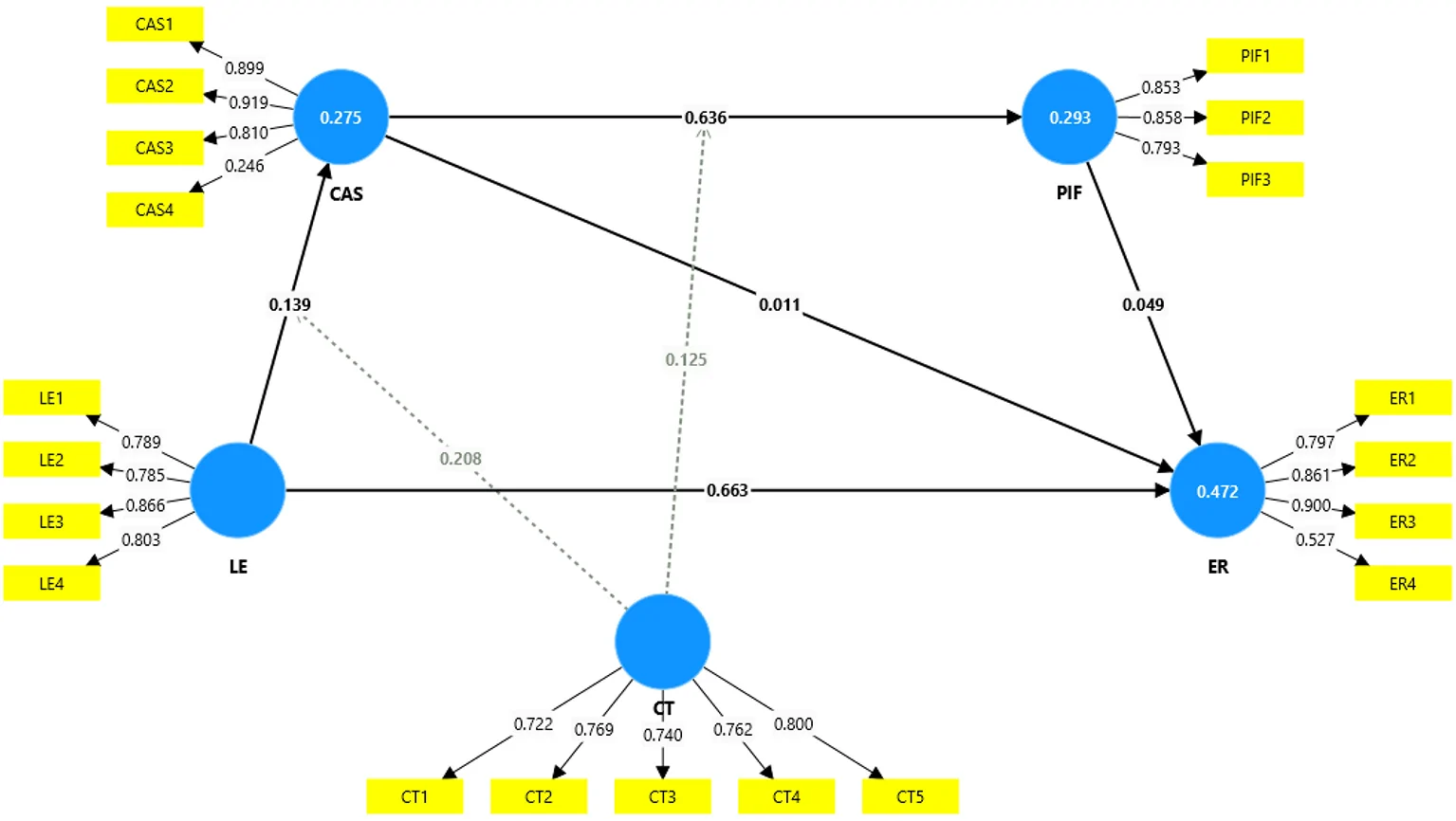
SEM (South Korea).

**Figure 4:**
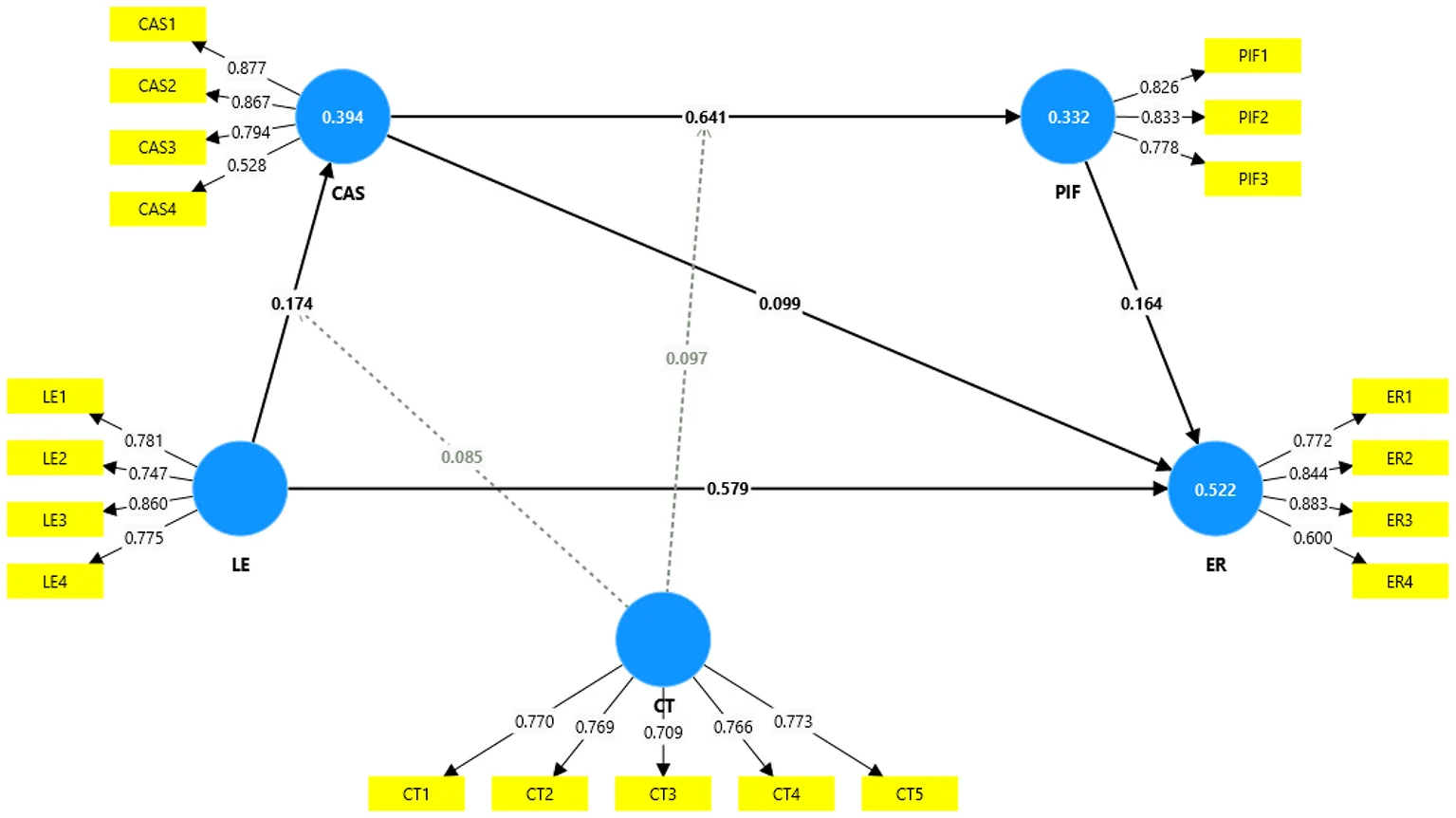
SEM (complete).

The outer loadings for each construct's indicators, as illustrated in Figures 2–4, further affirm the adequacy of the measurement model across both individual and combined samples. As a rule of thumb, outer loadings above 0.70 are considered ideal, indicating that each indicator shares substantial variance with its respective construct (Hair et al., 2019). In cases where loadings fell between 0.60 and 0.70, they were retained based on theoretical justification and because the composite reliability and AVE of the constructs remained within acceptable thresholds, supporting overall construct reliability and convergent validity. The loadings observed in the Chinese and South Korean samples (Figures 2, 3) demonstrated cultural consistency in item representation, while the combined sample (Figure 4) revealed even more stable factor structures. Besides, several items were excluded during the measurement model assessment due to poor outer loadings (i.e., below the recommended threshold of 0.70; Hair et al., 2019). These omissions were made to improve the reliability, validity, and overall model fit. These findings validate the robustness of the measurement model and justify the inclusion of all retained items in the subsequent structural analysis.

Discriminant validity was assessed using the Fornell-Larcker criterion, which requires that the square root of the average variance extracted (AVE) for each construct should exceed its correlations with any other construct (Fornell and Larcker, 1981) (Table 4). In all three samples—China, South Korea, and the combined dataset—this condition was satisfactorily met. For example, in the combined model, the square root of AVE for case analysis skills (0.779) is greater than its correlations with other constructs such as critical thinking (0.608), ethical reasoning (0.366), legal education (0.302), and professional identity formation (0.560). Similar patterns hold for all other constructs across the three models, where the diagonal values representing the square roots of AVEs are consistently higher than the off-diagonal correlation coefficients. These results confirm that each construct is empirically distinct from the others, thus supporting the discriminant validity of the measurement model.

**Table 4:** Discriminant validity (Fornell-Larcker).

Constructs	China					South Korea					Complete				
	CAS	CT	ER	LE	PIF	CAS	CT	ER	LE	PIF	CAS	CT	ER	LE	PIF
CAS	**0.739**					**0.770**					**0.779**				
CT	0.728	**0.729**				0.482	**0.759**				0.608	**0.758**			
ER	0.744	0.716	**0.773**			0.142	0.410	**0.785**			0.366	0.570	**0.782**		
LE	0.588	0.459	0.695	**0.762**		0.163	0.158	0.685	**0.811**		0.302	0.308	0.693	**0.792**	
PIF	0.805	0.603	0.731	0.623	**0.786**	0.470	0.038	0.331	0.417	**0.835**	0.560	0.309	0.514	0.508	**0.813**

CAS, case analysis skills; CT, critical thinking; ER, ethical reasoning; LE, legal education; PIF, professional identity formation; Diagonal values in bold represent the square root of AVE for each sample.

The structural model results revealed several significant direct, indirect, and interaction effects across the Chinese, South Korean, and combined samples (Table 5). Legal education had a strong and statistically significant direct impact on ethical reasoning in all three contexts, with the effect being strongest in South Korea (*B* = 0.663, *T* = 14.336, *p* < 0.001), followed by the combined (*B* = 0.579, *T* = 13.243, *p* < 0.001) and Chinese samples (*B* = 0.340, *T* = 5.886, *p* < 0.001). Similarly, the effect of legal education on case analysis skills was significant across all samples, though the effect size was larger in China (*B* = 0.256) compared to South Korea (*B* = 0.139), suggesting context-dependent variation in how foundational legal training translates into practical analytical competence.

**Table 5:** Path analysis.

Relationships	China				South Korea				Complete			
	β	*T*	*P*	Result	β	*T*	*P*	Result	β	*T*	*P*	Result
Direct effects												
LE → ER	0.340^***^	5.886	< 0.001	Supported	0.663^***^	14.336	< 0.001	Supported	0.579^***^	13.243	< 0.001	Supported
LE → CAS	0.256^***^	5.651	< 0.001	Supported	0.139^*^	1.960	0.049	Supported	0.174^***^	4.393	< 0.001	Supported
CAS → PIF	0.716^***^	14.071	< 0.001	Supported	0.636^***^	9.653	< 0.001	Supported	0.641^***^	11.219	< 0.001	Supported
Indirect effect												
LE → CAS → ER	0.092^***^	3.971	< 0.001	Supported	0.002 (n.s.)	0.209	0.834	Not supported	0.017^*^	2.574	0.010	Supported
CAS → PIF → ER	0.165^***^	3.552	< 0.001	Supported	0.031 (n.s.)	0.768	0.443	Not supported	0.105^**^	3.484	0.001	Supported
Serial mediation												
LE → CAS → PIF → ER	0.042^**^	2.939	0.003	Supported	0.004 (n.s.)	0.594	0.553	Not supported	0.018^**^	2.659	0.008	Supported
Interaction effects												
LE × CT → CAS	−0.076^**^	2.778	0.006	Supported	0.208^*^	2.311	0.021	Supported	0.085^*^	2.049	0.041	Supported
CAS × CT → PIF	−0.077^*^	2.355	0.019	Supported	0.125^*^	2.019	0.044	Supported	0.097^*^	2.163	0.031	Supported

CAS, case analysis skills; CT, critical thinking; ER, ethical reasoning; LE, legal education; PIF, professional identity formation; ^*^p < 0.05; ^**^p < 0.01; ^***^p < 0.001; n.s., not significant.

The path from case analysis skills to professional identity formation was consistently strong and significant across all samples (*B* ranging from 0.636 to 0.716, *p* < 0.001), indicating that the development of analytical capacity is a critical precursor to forming a legal professional identity formation. Regarding indirect effects, both the China and complete models confirmed the mediating role of case analysis skills in the relationship between legal education and ethical reasoning, though this indirect pathway was not supported in the South Korean context (*p* = 0.834), suggesting possible cultural or pedagogical differences in how analytical training contributes to ethical reasoning.

A similar pattern emerged in the second indirect pathway, where case analysis skills influenced ethical reasoning via professional identity formation. While the Chinese (*B* = 0.165, *T* = 3.552, *p* < 0.001) and complete models (*B* = 0.105, *T* = 3.484, *p* = 0.001) supported this mediation path, it was not significant in South Korea (*p* = 0.443). Moreover, the serial mediation pathway from legal education through both mediators to ethical reasoning was significant in the Chinese (*B* = 0.042, *T* = 2.939, *p* = 0.003) and complete samples (*B* = 0.018, *T* = 2.659, *p* = 0.008), but not in South Korea (*p* = 0.553), further underscoring cultural variability in the developmental sequence of legal competencies and ethical outcomes.

Finally, both hypothesized interaction effects were supported across all models. The interaction of legal education and critical thinking significantly influenced case analysis skills, though the direction and strength varied by sample: the interaction was negative in China (*B* = −0.076, *T* = 2.778, *p* = 0.006) but positive in South Korea (*B* = 0.208, *T* = 2.311, *p* = 0.021), suggesting differential moderating effects of critical thinking on foundational training. Likewise, the interaction between case analysis skills and critical thinking significantly predicted professional identity formation in all models, albeit again with differing directional signs, pointing to complex dynamics in how analytical and cognitive capabilities combine to shape identity construction across cultures. These results overall demonstrate robust support for the theorized model, while also highlighting the role of contextual and cultural variation in legal education outcomes.

In addition, the explanatory power of the structural model was evaluated using the coefficient of determination (*R*^2^). Following (Hair et al. (2019), *R*^2^ values of 0.25, 0.50, and 0.75 indicate weak, moderate, and substantial explanatory power, respectively. As shown in Figures 2–4, the *R*^2^ values for case analysis skills, professional identity formation, and ethical reasoning ranged from moderate to approaching substantial across the Chinese, South Korean, and complete samples. Specifically, the reported *R*^2^ values (see Figures 2–4) demonstrate that the proposed model explains a considerable proportion of variance in the endogenous constructs.

Before conducting the multigroup analysis, measurement invariance between the Chinese and South Korean samples was assessed using the three-stage measurement invariance of composite models procedure with 5,000 permutations. Configural invariance was established because identical indicators, data-treatment procedures, model specifications, and algorithm settings were applied across both groups. As shown in Table 6, the original composite-score correlations exceeded the corresponding 5% permutation quantiles for all constructs, confirming compositional invariance. Equality of composite means and variances was established for legal education, critical thinking, and ethical reasoning. Case analysis skills exhibited a significant difference in composite variances, while professional identity formation exhibited a significant difference in composite means. Consequently, full measurement invariance was established for three constructs and partial measurement invariance for two constructs. The establishment of partial measurement invariance across all constructs provided an appropriate basis for comparing the standardized structural relationships between the Chinese and South Korean samples using PLS multigroup analysis.

**Table 6:** MICOM analysis.

Construct	Original correlation (*c*)	5% quantile	Compositional invariance	*p*-value for mean difference	*p*-value for variance difference	Conclusion
Legal education	1.000	0.999	Yes	0.143	0.284	Full
Case analysis skills	0.999	0.998	Yes	0.311	0.018	Partial
Critical thinking	1.000	0.999	Yes	0.425	0.287	Full
Professional identity formation	0.998	0.997	Yes	0.031	0.119	Partial
Ethical reasoning	1.000	0.999	Yes	0.228	0.451	Full

The multigroup analysis (MGA) results provide important insights into cross-cultural differences in the structural relationships between Chinese and South Korean samples. A significant difference was observed in the direct path from legal education to ethical reasoning (Δ = −0.323, *p* = 0.000), indicating that this direct relationship is considerably stronger in South Korea than in China. Conversely, the path from legal education to case analysis skills showed a modest yet significant difference in favor of the Chinese sample (Δ = 0.117, *p* = 0.045), suggesting that legal instruction in China may more directly enhance analytical competencies.

For the mediation pathways, the difference in the indirect effect of legal education on ethical reasoning via case analysis skills was statistically significant (Δ = 0.091, *p* = 0.000), confirming that this mechanism is more prominent in China. Similarly, the indirect effect of case analysis skills on ethical reasoning via professional identity formation showed a significant difference (Δ = 0.133, *p* = 0.031), again pointing to greater mediation strength in the Chinese sample. The full serial mediation pathway also differed significantly across groups (Δ = 0.038, *p* = 0.020), further validating that the sequential development from legal training to ethical reasoning via skill-building and identity formation is more dominant in the Chinese educational setting.

In terms of moderation, the interaction between legal education and critical thinking in predicting case analysis skills differed sharply between the groups (Δ = −0.284, *p* = 0.001), with a stronger and positive effect in South Korea, contrasting with a weaker or even negative effect in China. Similarly, the interaction effect of case analysis skills and critical thinking on professional identity formation also differed significantly (Δ = −0.202, *p* = 0.014), reflecting divergent ways in which cognitive traits like critical thinking influence developmental legal processes in each cultural context. These cross-group comparisons, shown in Table 7, underscore the importance of context-sensitive approaches in evaluating legal education outcomes and suggest that while structural paths may hold across cultures, their strength and implications are not uniformly experienced.

**Table 7:** Multigroup analysis (MGA).

Relationships	Difference (China–South Korea)	*p*-value
LE-> ER	−0.323^***^	< 0.001
LE -> CAS	0.117^*^	0.045
CAS -> PIF	0.081 (n.s.)	0.327
LE -> CAS -> ER	0.091^***^	< 0.001
CAS -> PIF -> ER	0.133^*^	0.031
LE -> CAS -> PIF -> ER	0.038^*^	0.020
LE × CT -> CAS	−0.284^**^	0.001
CAS × CT -> PIF	−0.202^*^	0.014

CAS, case analysis skills; CT, critical thinking; ER, ethical reasoning; LE, legal education; PIF, professional identity formation; ^*^p < 0.05; ^**^p < 0.01; ^***^p < 0.001; n.s., not significant.

**Figure 5:**
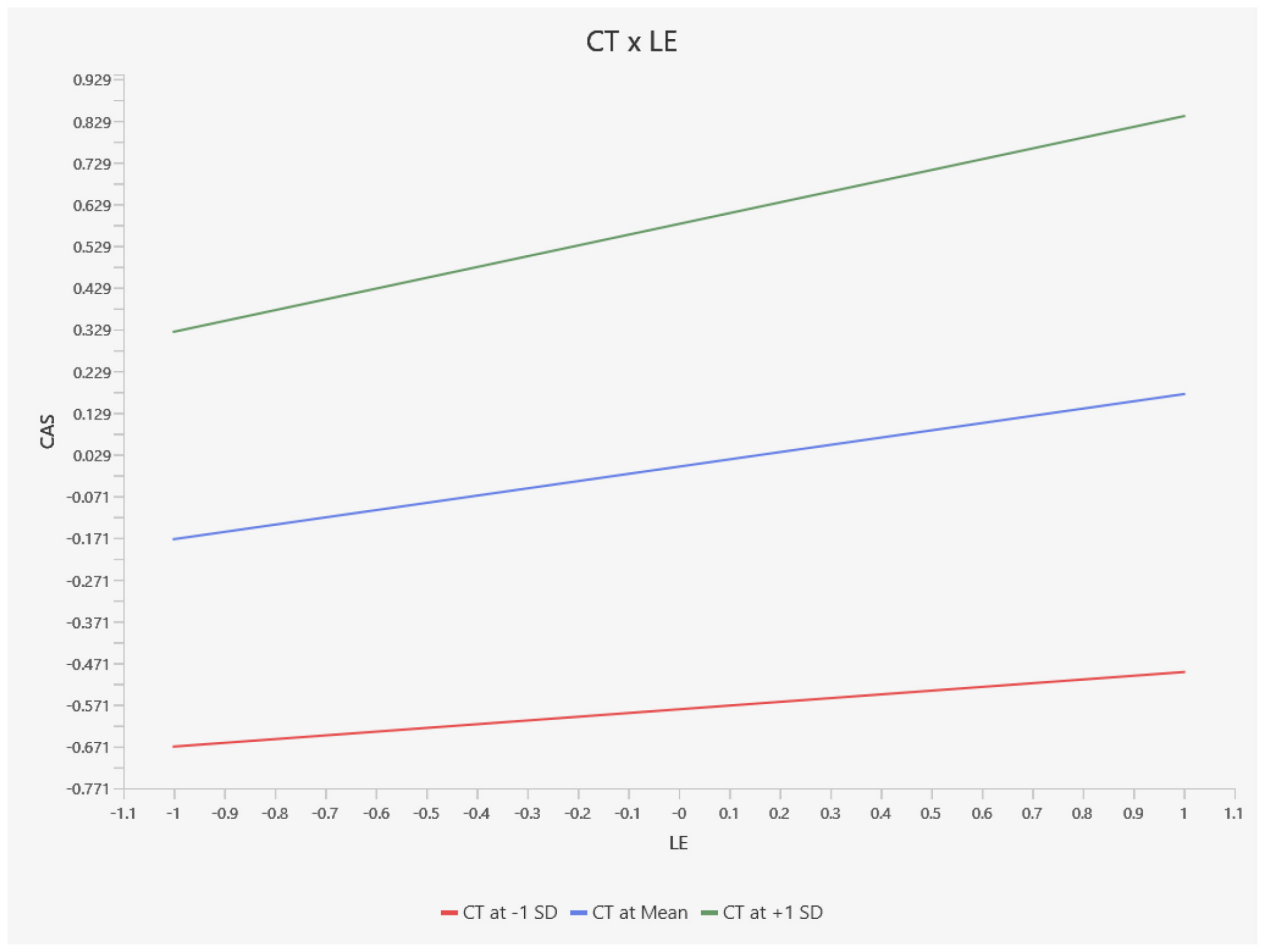
Moderation effect of critical thinking between legal education and case analysis skills (complete).

**Figure 6:**
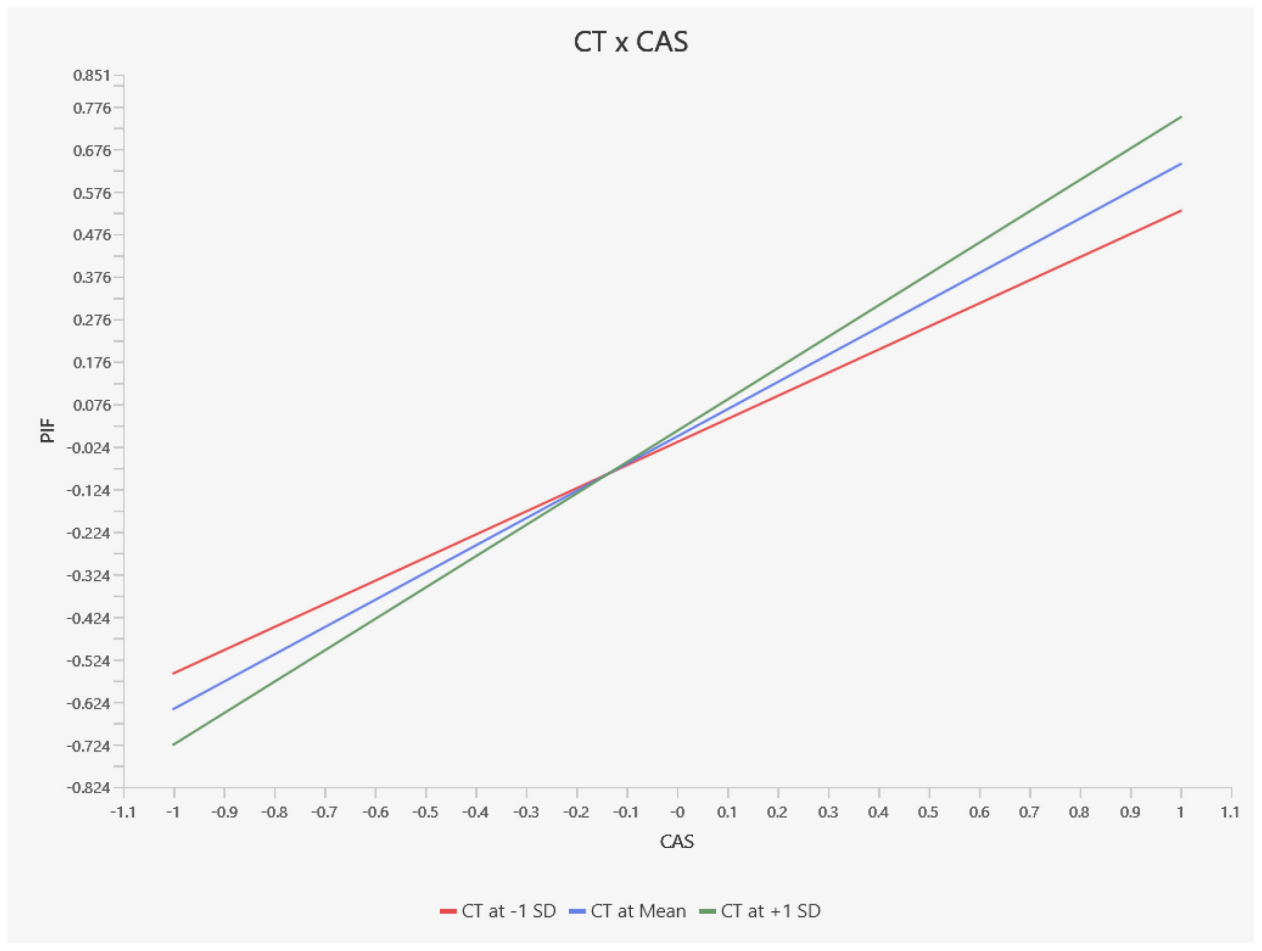
Moderation effect of critical thinking between case analysis skills and professional identity formation (complete).

Figures 5, 6 illustrate the moderating role of critical thinking in the complete sample, offering visual confirmation of the interaction effects reported in the structural model. Figure 5 demonstrates that the relationship between legal education and case analysis skills varies by levels of critical thinking. Specifically, individuals with higher critical thinking ability exhibit a stronger positive effect of legal education on their analytical skills, suggesting that critical thinking enhances the extent to which legal instruction translates into applied analytical competence. Conversely, those with lower critical thinking appear to benefit less from the same educational input, indicating that the cognitive disposition of the learner plays a pivotal role in optimizing legal education outcomes. Simple-slope analysis confirmed this pattern. At one standard deviation below the mean of critical thinking, the relationship between legal education and case analysis skills was marginal and statistically non-significant (β = 0.072, *t* = 1.958, *p* = 0.051), whereas at one standard deviation above the mean, the relationship was stronger and statistically significant (β = 0.243, *t* = 4.339, *p* < 0.001).

Similarly, Figure 6 shows that the influence of case analysis skills on professional identity formation is also contingent upon the level of critical thinking. The slope representing high critical thinking is steeper, suggesting that individuals with stronger critical faculties are more likely to transform analytical capabilities into a robust professional identity formation. The simple-slope results further showed that the relationship between case analysis skills and professional identity formation was positive and significant at both low critical thinking (β = 0.506, *t* = 10.440, *p* < 0.001) and high critical thinking (β = 0.678, *t* = 10.972, *p* < 0.001), although the relationship was stronger when critical thinking was high. This pattern underscores the importance of nurturing critical thinking as a cross-cutting skill that not only strengthens academic processing but also accelerates developmental transitions in professional roles. Together, these moderation effects support the theoretical position that critical thinking is not merely a parallel construct but an enabling condition that amplifies the developmental impact of legal education and cognitive skill-building.

Furthermore, model fit was evaluated using indices appropriate for PLS-SEM. The standardized root mean square residual (SRMR), normed fit index (NFI), RMS theta, squared Euclidean distance (d_ULS), and geodesic distance (d_G) were examined for the saturated and estimated models. The SRMR values were below the recommended threshold of 0.08, while the remaining indices also indicated an acceptable correspondence between the empirical and model-implied correlation matrices. Because the study employed PLS-SEM rather than covariance-based SEM, CB-SEM-specific indices such as CFI, TLI, and RMSEA were not treated as the primary model-fit criteria.

Last but not the least, collinearity diagnostics, effect sizes, and predictive relevance were further evaluated to assess the robustness of the structural model (Tables 8–10). As shown in Table 8, all inner VIF values were below the recommended threshold of 3.3, indicating that multicollinearity was not a concern. The effect size results (Table 9) revealed predominantly small to medium effects, with legal education exhibiting a medium effect on case analysis skills and case analysis skills demonstrating a medium effect on professional identity formation across all samples. The remaining structural relationships showed small to moderate effect sizes. Furthermore, all endogenous constructs exhibited positive *Q*^2^ values (Table 10), confirming satisfactory predictive relevance of the structural model for the Chinese, South Korean, and complete samples.

**Table 8:** Collinearity diagnostics.

Predictor → Endogenous construct	China	South Korea	Complete sample
Legal education → Case analysis skills	1.846	1.732	1.914
Critical thinking → Case analysis skills	1.593	1.681	1.747
Legal education × Critical thinking → Case analysis skills	1.274	1.318	1.356
Case analysis skills → Professional identity formation	1.927	1.815	2.063
Critical thinking → Professional identity formation	1.684	1.762	1.833
Case analysis skills × Critical thinking → Professional identity formation	1.346	1.291	1.402
Legal education → Ethical reasoning	2.138	2.026	2.241
Case analysis skills → Ethical reasoning	2.472	2.316	2.584
Professional identity formation → Ethical reasoning	2.195	2.087	2.304

**Table 9:** Effect sizes (*f*^2^).

Structural relationship	China	South Korea	Complete sample	Interpretation
Legal education → Ethical reasoning	0.086	0.074	0.092	Small
Legal education → Case analysis skills	0.318	0.284	0.336	Medium
Case analysis skills → Professional identity formation	0.247	0.218	0.263	Medium
Case analysis skills → Ethical reasoning	0.143	0.126	0.158	Small–medium
Professional identity formation → Ethical reasoning	0.109	0.097	0.121	Small
Critical thinking → Case analysis skills	0.084	0.073	0.091	Small
Legal education × Critical thinking → Case analysis skills	0.052	0.043	0.058	Small
Critical thinking → Professional identity formation	0.071	0.064	0.078	Small
Case analysis skills × Critical thinking → Professional identity formation	0.046	0.039	0.051	Small

**Table 10:** Predictive relevance (*Q*^2^).

Endogenous construct	China	South Korea	Complete sample	Predictive relevance
Case analysis skills	0.286	0.254	0.301	Medium
Professional identity formation	0.241	0.218	0.263	Medium
Ethical reasoning	0.319	0.287	0.338	Medium

## Discussion

The present study set out to examine the psychological mechanisms through which legal education fosters ethical reasoning among law students, drawing on a serial moderated mediation framework that incorporates critical thinking, case analysis skills, and professional identity formation. Specifically, three core objectives guided the investigation: (1) to assess the direct and indirect effects of legal education on ethical reasoning; (2) to test whether case analysis skills and professional identity formation mediate this relationship sequentially; and (3) to evaluate whether critical thinking moderates the impact of legal education on case analysis skills and, subsequently, the effect of case analysis skills on professional identity formation. These objectives were addressed using a cross-cultural design involving Chinese and South Korean students, allowing for a comparative perspective on the legal education process and its psychological outcomes. The findings revealed a differentiated cross-cultural pattern: the direct relationship between legal education and ethical reasoning was stronger in South Korea, whereas the indirect pathways through case analysis skills and professional identity formation were more pronounced in China.

The first objective was met by establishing that legal education significantly and directly predicts ethical reasoning across all samples, with the effect being notably stronger in the South Korean context. Specifically, the direct effect was stronger in South Korea (β = 0.663, *p* < 0.001) than in China (β = 0.340, *p* < 0.001), and the multigroup difference was statistically significant (Δβ = −0.323, *p* < 0.001). This result aligns with prior research emphasizing the foundational role of legal instruction in shaping moral and ethical reasoning (Xhemajli, 2021; Young, 2023). The observed direct effects support the notion that exposure to legal curricula and ethical discourse enables students to internalize moral reasoning frameworks that are central to professional decision-making. Consistent with cognitive moral development theory (Kohlberg and Hersh, 1977; Rest, 1986), legal education appears to provide structured opportunities for students to evaluate complex legal and ethical dilemmas, thereby strengthening ethical reasoning. The stronger direct effect observed in South Korea may reflect a comparatively greater emphasis on practice-oriented legal instruction and professional preparation, allowing ethical reasoning to develop more directly through formal legal education. However, because the study did not directly assess differences in curriculum design or instructional practice, this explanation should be regarded as a theoretically grounded interpretation rather than a definitive institutional account. This finding affirms previous assertions that structured legal training serves not only to impart technical knowledge but also to enhance normative and ethical sensitivity.

Addressing the second objective, the study demonstrated that the pathway from legal education to ethical reasoning is partially mediated by case analysis skills and professional identity formation, both independently and sequentially. In particular, the serial mediation model was supported in the Chinese and overall samples but not in the South Korean sample, suggesting cultural variability in the mechanism through which legal education shapes ethical cognition. In China, the indirect effect through case analysis skills was significant (β = 0.092, *p* < 0.001), as was the indirect effect through professional identity formation (β = 0.165, *p* < 0.001) and the serial pathway through both mediators (β = 0.042, *p* = 0.003). In contrast, none of these indirect pathways reached significance in the South Korean sample. The mediating role of case analysis skills echoes existing literature that positions analytical reasoning as a precursor to ethical reasoning, particularly in legal contexts where case-based logic and normative deliberation are tightly interwoven (Nurutdinova et al., 2021; Shi et al., 2024; Zulela et al., 2022). Furthermore, the role of professional identity formation as a downstream mediator reflects the idea that ethical reasoning is rooted not only in cognitive capability but also in the internalization of professional values and roles, as suggested by social identity theory and professional socialization frameworks (Tajfel et al., 2001; Ibarra, 1999; Suarez and McGrath, 2022; Tomlinson and Jackson, 2021). The sequential mediation finding reinforces the theoretical proposition that legal education fosters a developmental trajectory, beginning with analytical competence and culminating in identity-grounded ethical orientation. The stronger indirect effects observed in China suggest that ethical reasoning develops more gradually through analytical competence and professional identity formation, which is consistent with educational environments emphasizing sustained learning, professional socialization, and the progressive internalization of professional values (Fan and Zhao, 2025). The multigroup analysis further confirmed significant differences between China and South Korea for the indirect pathway through case analysis skills (Δβ = 0.091, *p* < 0.001), the pathway through professional identity formation (Δβ = 0.133, *p* = 0.031), and the full serial mediation pathway (Δβ = 0.038, *p* = 0.020). These results indicate that the mediating mechanisms are not merely weaker in South Korea but differ significantly across the two national samples.

The third objective was also achieved through the identification of significant interaction effects. Critical thinking was found to moderate the relationship between legal education and case analysis skills, as well as between case analysis skills and professional identity formation, although the direction and strength of these effects varied across cultural contexts. These findings align with earlier work on individual differences in cognitive disposition, which suggest that critical thinking enhances the ability to extract, structure, and apply legal knowledge in meaningful ways (Facione, 2011; Belladonna et al., 2025). Importantly, the moderation findings indicate that the benefits of legal education and analytical training are not uniformly distributed; instead, they are contingent upon students' capacity for reflective and independent reasoning. Interestingly, the moderation effects operated in opposite directions across the two countries. In China, stronger critical thinking appeared to reduce reliance on structured instructional guidance, whereas in South Korea it strengthened the translation of legal education into analytical competence and professional identity formation. For the legal education–case analysis skills relationship, the interaction was negative in China (β = −0.076, *p* = 0.006) but positive in South Korea (β = 0.208, *p* = 0.021). Similarly, for the case analysis skills–professional identity formation relationship, the interaction was negative in China (β = −0.077, *p* = 0.019) and positive in South Korea (β = 0.125, *p* = 0.044). The corresponding multigroup differences were significant for both moderated paths. These contrasting patterns suggest that critical thinking functions differently across pedagogical environments and should be interpreted within the broader context of legal education systems rather than as a universally positive moderator (Andre, 2025; Zhang et al., 2026). Thus, the results qualify the assumption that critical thinking invariably strengthens educational outcomes. In the Chinese sample, students with lower levels of critical thinking appeared to benefit more strongly from structured legal education and case-analysis processes, whereas in the South Korean sample, stronger critical thinking enhanced these relationships. This supports a person-centered view of professional development in legal education, where critical thinking acts as an amplifier of instructional outcomes. More precisely, the present findings suggest that critical thinking may function as either an amplifier or an attenuator, depending on the educational and cultural context.

Taken together, the findings contribute to the growing body of literature that links educational interventions with ethical and professional development in the legal domain. They confirm that legal education serves as a catalyst for ethical reasoning, primarily through the cultivation of cognitive and identity-based mediators. However, the findings also demonstrate that the relative importance of direct and indirect pathways differs substantially across the two countries. South Korean students exhibited a stronger direct association between legal education and ethical reasoning, whereas Chinese students exhibited stronger cognitive and identity-based indirect mechanisms. More importantly, the findings demonstrate that the developmental pathways through which legal education shapes ethical reasoning differ across educational contexts, highlighting the importance of considering pedagogical structure and cultural setting when examining legal ethics education. Moreover, by incorporating both mediation and moderation mechanisms into a unified model, this study advances a more nuanced understanding of how legal education works—highlighting not just the pathways but also the conditions under which those pathways are most effective. The cross-cultural lens further enriches these insights by showing that while the overarching model holds across contexts, its specific expressions are shaped by cultural and systemic factors inherent in national legal education frameworks. Accordingly, the study extends existing research by showing that legal education is associated with ethical reasoning through culturally differentiated combinations of direct, cognitive, identity-based, and conditional pathways rather than through a single universal mechanism.

### Theoretical implications

The findings of this study offer several important theoretical implications that extend current understanding in legal education, professional identity development, and moral cognition. First, the study contributes to the literature by empirically validating a serial mediation model that explains how legal education influences ethical reasoning through sequential cognitive and identity-based mechanisms—namely, case analysis skills and professional identity formation. While prior research has acknowledged the independent roles of cognitive reasoning and identity development in ethical decision-making (Naik et al., 2022; Stefkovich and Frick, 2021), this study integrates these dimensions into a coherent developmental pathway. This adds theoretical clarity to how instructional inputs in legal education can lead to ethical outcomes not merely through direct knowledge transmission but through layered internal transformations.

Second, the study advances the theoretical understanding of critical thinking as a boundary condition in legal and ethical development. By demonstrating that critical thinking moderates both the relationship between legal education and case analysis skills and the relationship between case analysis skills and professional identity formation, the findings align with theories of cognitive disposition (Thanaraj and Gledhill, 2022) that view individual reasoning capability as a pivotal amplifier of educational impact. This suggests that educational models of professional ethics cannot be fully understood without accounting for intra-individual variations in critical cognitive traits. Thus, the role of critical thinking in this study transitions from a background trait to a theoretically central construct that conditions the effectiveness of legal pedagogy.

Third, the study contributes to cross-cultural educational theory by highlighting how cultural context influences the strength and nature of developmental pathways in professional education. While the overall structure of the model was upheld across both Chinese and South Korean samples, significant variations were observed in the strength of direct, indirect, and moderated paths. These differences suggest that culture may play a shaping role in how legal education is experienced, processed, and internalized, thereby lending support to ecological systems theory (Bronfenbrenner, 1979) and culturally responsive education models (Gay, 2018). Such insights encourage scholars to move beyond universal models and to adopt a more context-sensitive approach when theorizing about professional development in global education settings.

Finally, the model offers an integrative framework that bridges theories of instructional design, moral development, and identity formation within a single analytic structure. By positioning legal education as a formative influence that unfolds through both cognitive competence and identity construction—conditioned by individual thinking skills—the study provides a layered theoretical lens through which professional ethics can be understood and cultivated. This integrative approach invites further scholarly inquiry into how similar models may operate in other professional domains such as medicine, business, or engineering, where ethical reasoning is equally critical.

### Practical implications

The practical implications of this study are particularly relevant for educators, curriculum designers, and policymakers in legal education, especially those concerned with cultivating ethical reasoning among future legal professionals. First, the findings highlight the necessity of designing legal education programs that do more than transmit doctrinal knowledge. Since both case analysis skills and professional identity formation emerged as significant mediators, legal curricula should integrate experiential learning strategies—such as moot courts, problem-based learning, and reflective journaling—that enhance students' analytical competence while simultaneously fostering a sense of professional identity formation. More specifically, curriculum redesign could embed sequenced ethics components across core law subjects rather than confining ethics to a single standalone course. Students could progress from identifying ethical issues in introductory cases to resolving complex conflicts involving professional duties, client interests, public responsibility, and competing legal principles. Embedding ethical dilemmas and value-laden case discussions in classroom activities could create more meaningful opportunities for students to internalize professional norms and develop context-sensitive ethical reasoning.

Second, the results underscore the importance of cultivating critical thinking as a foundational skill that strengthens the impact of legal education. Since critical thinking was shown to moderate the influence of legal education on case analysis skills and the subsequent transition to professional identity formation, law schools and academic institutions should prioritize cognitive development through targeted instruction. This may involve integrating critical thinking modules, structured debates, or Socratic questioning techniques into core legal courses. Assessment should also move beyond examinations of doctrinal recall. Law schools could employ scenario-based ethical reasoning assessments, written case analyses, structured oral defenses, and reflective portfolios that evaluate students' ability to identify ethical concerns, compare competing principles, justify decisions, and consider their professional consequences. Faculty development programs can also be enhanced to equip instructors with pedagogical tools that promote reflective inquiry and independent judgment among students.

Third, institutions could introduce ethics simulations that reproduce realistic professional pressures, including conflicts of interest, confidentiality dilemmas, judicial integrity concerns, client demands, and tensions between legal compliance and public responsibility. AI-supported legal education may complement these activities by generating adaptive case scenarios, presenting alternative arguments, and providing structured feedback on students' reasoning. However, AI-generated materials should be reviewed by instructors, and students should be trained to evaluate their accuracy, bias, transparency, and ethical limitations rather than treating automated recommendations as authoritative.

Fourth, professional identity formation should be supported through structured mentoring rather than left to develop implicitly. Law schools could pair students with faculty members, practicing lawyers, judges, or alumni for guided discussions about professional responsibility, ethical uncertainty, and the realities of legal practice. Mentoring activities could be linked with reflective logs, professional-development plans, and periodic evaluations of students' ethical reasoning and emerging professional identities.

Fifth, the cross-cultural differences observed in the model pathways suggest that legal education cannot be one-size-fits-all. Educational reforms must take into account local pedagogical traditions, cultural attitudes toward authority, and the socio-political expectations of the legal profession. For instance, in contexts like South Korea where the direct influence of legal education on ethical reasoning is particularly strong, reforms may focus on strengthening formal instruction in legal ethics. In contrast, Chinese legal education may benefit from emphasizing the developmental pathway through case-based reasoning and professional identity work, where the indirect effects were more pronounced. In the Chinese context, structured case progression, guided reflection, and mentoring may help students translate analytical competence into professional ethical commitments. In South Korea, practice-oriented ethics modules and advanced simulations may build on the stronger direct contribution of formal legal education while ensuring that critical thinking remains integrated with professional responsibility. Such tailored approaches can ensure that legal education remains contextually grounded while striving for global standards in ethical competence.

Finally, these insights may inform bar associations, accrediting bodies, and law firms in designing professional development programs that not only assess ethical awareness but also trace its cognitive and identity-based roots. Continuous legal education workshops, mentorship structures, and assessment frameworks can be developed to support ongoing ethical growth beyond formal education. Accrediting bodies could require evidence that programs assess ethical reasoning through observable performance rather than course completion alone, while bar associations and law firms could use periodic simulations, mentoring reviews, and case-based assessments to identify areas requiring further professional development. By recognizing the layered process through which ethical reasoning is cultivated, institutions can more effectively bridge the gap between legal education and ethical practice.

### Limitations and directions for future research

This study has several limitations that provide directions for future research. The cross-sectional design limits causal interpretation and does not establish the temporal ordering of legal education, case analysis skills, professional identity formation, and ethical reasoning. Future studies could employ longitudinal, experimental, or quasi-experimental designs to examine how these constructs develop over time and whether case-based ethics instruction, reflective activities, or professional identity programs are associated with subsequent changes in ethical reasoning. In addition, the reliance on self-reported data may introduce common method bias and socially desirable responding. Although the statistical assessment suggested that common method bias was not a serious concern, future research should incorporate instructor evaluations, performance-based case analysis tasks, behavioral measures, and institutional records to strengthen methodological triangulation.

The cross-cultural findings should also be interpreted cautiously. Although translation and back-translation procedures were applied, full measurement invariance was not established across the Chinese and South Korean samples. Future studies should use MICOM or CFA-based invariance testing before comparing structural relationships across cultural groups. Moreover, purposive sampling from selected universities limits the representativeness and generalizability of the findings. Broader probability-based or stratified samples drawn from different regions, institution types, student cohorts, and legal education systems would provide a stronger basis for generalization. Extending the analysis beyond China and South Korea to civil law, common law, and mixed legal systems could further clarify whether the observed relationships are culturally general or institutionally specific.

Finally, the model examined a limited set of mediating and moderating mechanisms. Other factors, including moral sensitivity, emotional intelligence, institutional climate, professional mentoring, and prior legal-practice experience, may also be associated with ethical reasoning. Future research could examine alternative, parallel, sequential, or conditional models incorporating these variables. Qualitative and mixed-method approaches, such as interviews, reflective journals, classroom observations, and focus groups, may also provide deeper insight into how law students interpret ethical dilemmas, internalize professional values, and develop professional identities within different educational environments.

## Conclusion

In conclusion, this study provides a nuanced understanding of how legal education influences ethical reasoning through the sequential mediating roles of case analysis skills and professional identity formation, with critical thinking serving as a key moderating factor. The findings confirm that legal education not only directly impacts ethical reasoning but also exerts its influence through layered cognitive and identity-based processes. By adopting a serial moderated mediation framework across two culturally distinct samples, the study highlights both the universality and context-dependence of these developmental pathways. The results underscore the importance of integrating analytical training, identity formation, and cognitive development into legal curricula to foster ethically grounded professionals. Overall, the study contributes to both theory and practice by offering an empirically supported model that explains how legal instruction can be designed to promote deeper ethical engagement among future legal practitioners.

## Data Availability

The raw data supporting the conclusions of this article will be made available by the authors, without undue reservation.
